# Supplementary material for: Propolis as a Key Source of p-Coumaric Acid Permeating Honey and Sucrose Syrup Stores of Honey Bees
Source: Insects. 2025 Nov 13;16(11):1159. doi: 10.3390/insects16111159 (PMC12653071; doi:10.3390/insects16111159)
Supplement: Supplementary file 1 [file insects-16-01159-s001.zip › insects-3933722-supplementary.pdf]

SUPPLEMENTARY MATERIAL

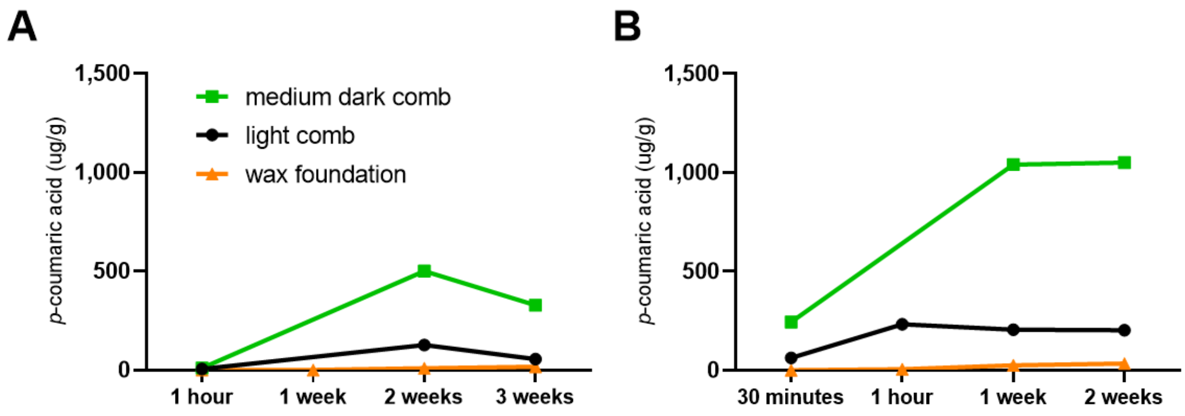

Figure S1: The extraction of *p*-coumaric acid from empty storage combs. Comparing the efficiency of *p*-coumaric acid extraction from the same wax combs using water (**A**) or methanol (**B**). Combs of various colours built from wax foundations were analyzed.

Apiary number	Name of location	GPS coordinates
1	Týn nad Vltavou	49.2396380 N, 14.4126577 E
2	Sedlce	48.8273597 N, 14.4927964 E
3	Doubravice	48.9391814 N, 14.5143854 E
4	České Budějovice 7, Plavská	48.9593260 N, 14.4763329 E
5	České Budějovice 31, town hall	48.9743123 N, 14.4731247 E
6	České Budějovice 5, Biology centre	48.9756609 N, 14.4459708 E
7	České Budějovice 5B, Na Sádkách	48.9750381 N, 14.4573120 E

Table S1: **GPS coordinates of apiaries sampled in this study.**
